# Exploring factors associated with increased suicides during the COVID‐19 pandemic in Japan: A study using data from postmortem examinations conducted in an urban area

**DOI:** 10.1002/pcn5.70005

**Published:** 2024-09-01

**Authors:** Yotaro Katsumata, Toshiaki Okano, Tadashi Takeshima, Yuka Igarashi

**Affiliations:** ^1^ Department of Human and Social Sciences Tokyo Metropolitan University Tokyo Japan; ^2^ Okano Medical Clinic Kawasaki Japan; ^3^ Kawasaki City Inclusive Rehabilitation Center Kawasaki Japan

**Keywords:** suicide, COVID‐19, multiple mental disorders, postmortem examinations

## Abstract

**Aim:**

The purpose of this study is to examine factors associated with increased suicide deaths during the coronavirus disease 2019 (COVID‐19) pandemic in Japan using primary data from postmortem examinations.

**Methods:**

We explored factors associated with suicides that occurred during the COVID‐19 pandemic (February 2020 to December 2021) using data from 115 postmortem examinations of suicides that occurred in one city in the Kanto region between January 2017 and December 2021.

**Results:**

Multivariate analysis using graphical modelling and logistic regression analysis showed that both female sex (adjusted odds ratio: 3.732; 95% confidence interval: 1.044–13.345) and multiple mental disorders (adjusted odds ratio: 7.344; 95% confidence interval: 1.316–40.987) were significantly associated with suicide during the COVID‐19 pandemic among the young age group (39 years or under).

**Conclusion:**

The study results suggest that in addition to the factor of female sex previously identified, morbidity due to multiple mental disorders may be associated with the increased suicides in Japan during the COVID‐19 pandemic. Furthermore, this study presented the new methodological possibility of analyzing background factors of suicide using postmortem examination data. In preparation for similar emergencies in the future, it is necessary to establish a system that provides care for multiple mental disorders and a continuous suicide‐monitoring system that combines methods such as psychological autopsies with other methods.

## Graphical abstract

In this study, we examined factors associated with increased suicide deaths during a coronavirus infection 2019 (COVID‐19) pandemic in Japan using primary data from postmortem examinations. The analysis suggested that in addition to the factor of female sex previously identified, morbidity due to multiple mental disorders may be associated with the increased suicides in Japan during the COVID‐19 pandemic.

## INTRODUCTION

Since 2020, novel coronavirus infections have been raging around the world, causing not only direct physical harm but also adversely affecting economies, people's lifestyles, and mental health.^1^ According to previous reports, although increases in suicidal ideation, self‐harm and suicide attempts were widely observed worldwide during the coronavirus disease 2019 (COVID‐19) pandemic,^2^, ^3^, ^4^ few countries reported a significant increase in suicide deaths.^5^

Japan is one of the few countries worldwide where a significant increase in suicide deaths was detected during the COVID‐19 pandemic. In Japan, seven major waves of COVID‐19 infections were observed until September 2022, with suicide rates increasing sharply in the second wave (June to October 2020), especially among young people and women.^6^ As of 2023, suicide rates for youth and women had not returned to pre‐pandemic levels.

In October 2022, given the impact of the spread of COVID‐19 infection, the Japanese government approved the Comprehensive Measures to Prevent Suicide, which represent strengthened measures against suicide among children, young people, and women. However, the background to the increase in suicides among young people and women during the COVID‐19 pandemic has not yet been fully clarified, and measures are being implemented with limited evidence.

Globally, mental health among youth and women was the most negatively affected by the COVID‐19 pandemic,^7^, ^8^ and Japan was no exception.^9^ Although severe behavioral restrictions or legal measures were not imposed in Japan during the pandemic, repeated mild lockdowns made it difficult for young people to attend school or work and contributed to isolation and loneliness, resulting in negative mental health effects.^10^ In addition, previous studies have found that low income, care for family members, domestic violence, fear of COVID‐19, and COVID‐19‐related stigma were associated with psychological stress among Japanese women during the pandemic.^11^, ^12^ Furthermore, information about a series of celebrity suicides in Japan during the pandemic spread through the mass media and the Internet—mainly social networking services—potentially causing the Werther effect.^11^

These intricately interrelated factors are assumed to be the backdrop of the increase in suicides among young people and women during the COVID‐19 pandemic. However, few studies have included data on suicide deaths, and these studies have focused on the analysis of macro data rather than data on individual cases.

In Japan, official suicide statistics are compiled and published by two agencies: the National Police Agency and the Ministry of Health, Labour, and Welfare. Statistics from the National Police Agency are recorded by place of discovery and are based on information from police investigations and all suicides in Japan, including those of foreigners. Statistics from the Ministry of Health, Labour, and Welfare are based on official death certificates or postmortem certificates prepared by doctors, are compiled by place of residence, and do not include data on foreigners. Although the number of suicides by sex, age, region, and means are available in both databases, more detailed information, such as occupation before death and the causes and motives of suicide, is available only from the National Police Agency; these data are often emphasized in analyses for policy‐making. However, these published statistical data are merely secondary data compiled by the police based on their own judgement of the information obtained in the investigation process; therefore, the results of analyses using such macro data are likely to contain considerable bias. In addition, it has also been pointed out that the statistics from the National Police Agency may contain data that are not suitable for annual comparison because the classification of occupation and the classification of causes and motives for suicide have been revised in the past.^13^

Although many neurological and physiological studies of suicide have been based on primary data collected by forensic medicine using samples collected at autopsy (e.g., Furczyk et al.^14^), studies that explore the psychosocial factors of suicide are uncommon outside of the few countries in which investigations such as psychological autopsies are systematically incorporated into forensic procedures. The death inquiry system varies widely across countries and regions, and the Japanese system takes limited account of the goal of preventing future loss of life under the same circumstances in which death occurred.^15^ Furthermore, given that forensic resources in Japan are scarce compared with those of other developed countries and psychological autopsies are not systematically incorporated into the postmortem examination process,^16^ the accuracy of psychosocial factor analysis in suicide based on information collected in postmortem examinations is relatively low.

However, this insufficient Japanese postmortem examination system is not completely without merit in suicide research. For example, because all deaths in Japan are examined, the use of these data has the advantage of minimizing sampling bias.^17^ In addition, the faster completion of the postmortem examination process in Japan compared with other countries may be useful for analysis and policy‐making given the urgent and rapidly changing nature of unusual deaths (such as the rapidly increasing suicide rate observed during a pandemic).

Except for a few case reports, no studies have been conducted to date in Japan using primary data on cases of suicide death during the COVID‐19 pandemic. Therefore, we compared data on suicide deaths before and during the COVID‐19 pandemic using the primary data from which the National Police Agency's statistical data are derived. Specifically, postmortem certificates and records of police investigations that were provided to doctors at the time of postmortem examinations were used to explore factors related to the increase in suicide deaths in Japan during the COVID‐19 pandemic.

## METHODS

### Data collection

The study was conducted using data from the postmortem examinations by the second author (T.O.) of cases in which the cause of death was classified as suicide during the 5 years from January 1, 2017 to December 31, 2021. Of a total of 118 cases collected, 115 cases were included in the analysis after excluding three cases in which the sex and age were unknown.

In Japan, “unusual deaths” are reported to the police in accordance with the Medical Practitioners' Act. A coroner's inspection (*kenshi* in Japanese) is conducted by a public prosecutor, a public prosecutor's assistant, or a judicial police officer to determine criminality in accordance with the Code of Criminal Procedure. The postmortem examination (*kenan* in Japanese) is the duty of a physician under the Medical Practitioners' Act and is performed after the coroner's inspection. In the postmortem examination, a police physician or general clinician makes a medical judgement on the cause of death, time since death, and other factors; based on the results, a postmortem certificate is prepared. As previously mentioned, as with the death certificate, which is prepared by a physician or dentist when the death is not unusual, the postmortem certificate is a document that medically and legally certifies the death of a human being and serves as a basic document for compiling statistics on the causes of death in Japan.

The second author (T.O.) is a general clinician who practices in an urban area with a population of more than 1.5 million in the Kanto region of Japan. As a physician who cooperates with the police, T.O. conducts postmortem examinations of unusual deaths that occur in his practice area, provides postmortem certificates to bereaved families as part of his duties, and maintains a database of the records of the postmortem examinations in which he is involved. In this database, in addition to the diagnostic details described in postmortem certificates, information from investigation records that are provided by police who conduct postmortem examinations is stored in text format, and the proper names of locations and persons are anonymized.

During the data‐collection period of this study, no major protocol changes were made to prevent infectious diseases in the postmortem examinations that involved the second author. Even during the COVID‐19 pandemic, postmortem examinations were conducted in the mortuaries of police stations, as in normal times. In cases in which COVID‐19 positivity was confirmed in advance, the body was masked to prevent the spread of residual expired breath, and the persons attending postmortem examinations wore personal protective equipment, including N95 masks. Although the second author did not conduct all of the postmortem examinations in the area and data on postmortem examinations in the area before and after the COVID‐19 pandemic were unavailable, no significant increase in the number of postmortem examinations was observed in 2020 and 2021 compared with earlier years, according to the results published by the neighboring Tokyo Metropolitan Government.^18^

### Data encoding

Because all of the data used in this study except for sex and age were recorded in the database in anonymized text format, coding was performed based on the consensus of several researchers (K.Y., T.T., and I.Y.) after the criteria were defined.

Regarding the presence or absence of mental disorder before death, we coded the presence of some form of mental disorder if both a history of psychiatric consultation and a psychiatric diagnosis were listed in the text data. Similarly, if multiple psychiatric diagnoses were listed together with a history of psychiatric examinations, the deceased was coded as having multiple mental disorders.

If a specific diagnosis of a physical disease was mentioned in the text data, the deceased was coded as having a history of physical disease.

In addition, we coded the deceased as having past suicidal behavior if any of the following were identified in the text data: expression of suicidal ideation to others before death, history of suicide attempts, or history of self‐injurious behavior.

The deceased was coded as having economic problems if either of the following were noted in the text data: late payment of rent or utility bills or “had financial difficulties.”

We identified from the text data and coded whether the respondents lived alone or with someone else. In four cases, identifying whether the respondents lived alone or with someone else was not possible; these cases were treated in the analysis as having missing values.

### Statistical analysis

In this study, we first defined the period before COVID‐19 as January 2017 to January 2020 and the period during COVID‐19 as February 2020 to December 2021. We compared the proportions of suicides that occurred within each period by sex, age group, a history of mental disorders before death, a history of physical illness before death, a history of suicidal behavior, economic problems, and residential status at death, that is, whether the patient lived alone or not.

Subsequently, we conducted a study using graphical modelling (GM) to exploratorily clarify the relationships between each variable. GM is a statistical multivariate analysis method for analyzing causal relationships and exploratorily modelling the causal relationships of variables based on data. We used the log‐linear graphical modelling (L‐GM) programme^19^ to explore undirected interactions among qualitative variables. A reduced model (RM) was evaluated objectively with deviances and *p*‐values using a backward elimination method with the full model (FM) and was presented as an independent graph model with edges and lines.

Finally, the factors associated with suicide during the COVID‐19 pandemic that were identified using GM were examined using logistic regression analysis and were stratified by age group.

All statistical analyses except the GM were performed using HAD,^20^ a free statistical software.

## RESULTS

A chi‐square test indicated that sex (*p* = 0.049), multiple mental disorders (*p* = 0.007), and economic problems (*p* = 0.018) were significantly associated with suicide during the COVID‐19 pandemic (Table 1).

**Table 1:** Association of each variable with suicide death before and during COVID‐19 pandemic.

	Before COVID‐19 (1/2017–1/2020)		During COVID‐19 (2/2020–12/2021)		*χ* ^2^	*p*	*Cramer's V*
	*n* = 63		*n* = 52				
	*n*	%	*n*	%			
Sex							
Male	44	69.8	27	51.9	3.872	0.049	0.183
Female	19	30.2	25	48.1			
Age group (years)							
<20	3	4.8	2	3.9	7.121	0.310	0.249
20–29	12	19.1	13	25.0			
30–39	11	17.5	10	19.2			
40–49	13	20.6	11	21.2			
50–59	2	3.2	5	9.6			
60–69	15	23.8	4	7.7			
>69	7	11.1	7	13.5			
Mental disorder							
Mental disorder of any kind	28	44.4	23	44.2	0.001	0.982	0.002
Multiple mental disorders	3	4.8	11	21.2	7.159	0.007	0.250
Physical illness	18	28.6	16	30.8	0.066	0.797	0.024
Past suicidal behavior	22	34.9	25	48.1	2.040	0.153	0.133
Economic problems	17	27.0	5	9.6	5.555	0.018	0.220
Living alone	20	32.8	21	42.0	1.001	0.317	0.095

**Figure 1:**
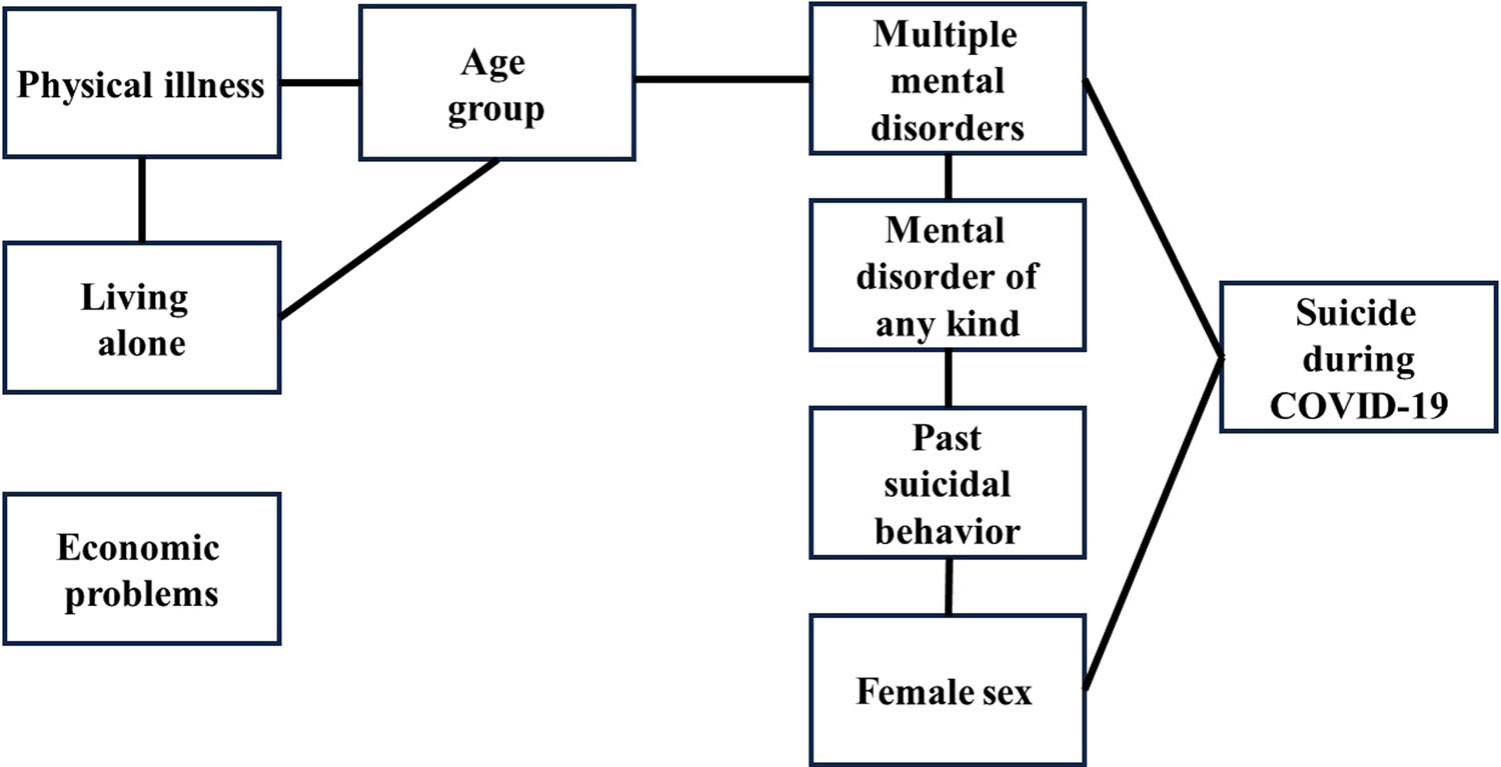
Independent graph of the final model.

Then, to identify a model that explained suicide during the COVID‐19 pandemic from the variables used in this study, we analyzed the associations between the variables using the L‐GM. After the creation of an undirected graph of the FM that assumed that all of the input variables were related, the paths of interaction between the factors were disconnected in the order of the *p*‐value of deviance, and the RM in which all paths were significant (*p* < 0.05) was adopted as the final model (Table 2). When the analysis was controlled for age, the results of the L‐GM showed that female sex and multiple mental disorders directly interacted with suicide during the COVID‐19 pandemic (Figure 1).

**Table 2:** *p*‐values and deviances of the final graphical modeling.

	1	2	3	4	5	6	7	8	9
1. Suicide during COVID‐19	****	0.043	―	―	0.006	―	―	―	―
2. Sex	4.090	****	―	―	―	―	0.000	―	―
3. Age group	―	―	****	―	0.004	0.001	―	―	0.001
4. Mental disorder of any kind	―	―	―	****	0.000	―	0.004	―	―
5. Multiple mental disorders	7.514	―	19.377	24.183	****	―	―	―	―
6. Physical illness	―	―	32.962	―	―	****	―	―	―
7. Past suicidal behavior	―	16.824	―	8.224	―	―	****	―	0.018
8. Economic problems	―	―	―	―	―	―	―	****	―
9. Living alone	―	―	31.940	―	―	16.975	―	―	****

The lower left diagonal of the table (left triangle) shows deviances, and the right upper (right triangle) shows *p*‐values in the table of the final model.

Given that all of the individuals were 39 years of age or under when we verified the relationship between multiple mental disorders and age group during the COVID‐19 pandemic (Table 3), we limited our analysis to younger individuals (39 years or under) and performed logistic regression analyses with sex and multiple mental disorders as independent variables and suicide during the COVID‐19 pandemic as the dependent variable. The results showed that both female sex (adjusted odds ratio: 3.732; 95% confidence interval: 1.044–13.345) and multiple mental disorders (adjusted odds ratio: 7.344; 95% confidence interval: 1.316–40.987) were significantly associated with suicide during the COVID‐19 pandemic (Table 4).

**Table 3:** Percentages of people with multiple mental disorders by age group.

Age group (years)	Before COVID‐19 (1/2017–1/2020)		During COVID‐19 (2/2020–12/2021)	
	*n*	%	*n*	%
<20	1	33.3	0	0.00
20–29	0	0.00	7	63.6
30–39	1	33.3	4	36.4
40–49	0	0.00	0	0.00
50–59	0	0.00	0	0.00
60–69	1	33.3	0	0.00
>69	0	0.00	0	0.00

**Table 4:** Factors associated with suicide during the COVID‐19 pandemic (under 39 years of age).

	*β*	Adjusted odds ratio [95% CI]
Sex		
Male	0.299*	(Reference)
Female		3.732 [1.044–13.345]
Multiple mental disorders		
No diagnosis	0.397*	(Reference)
With diagnosis		7.344 [1.316–40.987]

Abbreviation: 95% CI, 95% confidence interval.

* *p* < 0.05.

## DISCUSSION

Based on an analysis using data from postmortem examinations, this study found that female sex and multiple mental disorders in Japan were significantly associated with the sharp increase in suicides during the COVID‐19 pandemic when controlling for age (39 years or under). Although previous studies have reported a sharp increase in suicides among young people and women in the early stages of the COVID‐19 pandemic,^6^ this study suggests that multiple mental disorders are also associated with this sharp increase in the early stages of the pandemic.

In this study, the timing of the onset of multiple mental disorders was not specified; therefore, it is unclear whether the suicides were attempted by people who originally had multiple mental disorders that further worsened during the COVID‐19 pandemic or by those who developed multiple mental disorders during the COVID‐19 pandemic, which led to suicide. However, we can infer from previous studies that both processes likely occurred. For example, Hawton et al.^21^ found that approximately 50% of patients who had self‐harmed visited a hospital for worsened self‐harm during the COVID‐19 lockdown in England between March and May 2020, suggesting the worsening of an existing mental disorder or the influence of a newly developed mental disorder. In addition, physical distancing during the pandemic may have reduced available face‐to‐face support resources, including psychiatric care,^22^ and increased the difficulty in adopting daily stress coping strategies,^21^ potentially exacerbating existing mental disorders or leading to the development of new mental disorders. The increase in non‐face‐to‐face services during the COVID‐19 pandemic was a positive change.^22^, ^23^ However, the results of this study suggest that such resources alone are unlikely to prevent suicides among young people with a background of multiple mental disorders. The government's measures to date have not included countermeasures against multiple psychiatric disorders. Further research and the expansion of psychiatric resources are needed to establish effective treatment methods and prevent multiple psychiatric disorders during an infectious disease pandemic in Japan.

Although the presence of economic problems was not significantly associated with suicide during the COVID‐19 pandemic in the multivariate analysis of this study, the proportion of those with economic problems was significantly higher before the COVID‐19 pandemic than during the pandemic in the univariate analysis using the chi‐square test. Therefore, it is possible that the people in this study had more economic problems before the pandemic than during the pandemic. However, pre‐pandemic suicides in this study notably included a high proportion of men and older adults. Previous research has shown that male suicides are generally associated with more financial problems than are female suicides.^24^ Older adults also tend to be at an economically nonproductive age and are more likely than younger adults to have economic problems. In other words, differences in sex and age of suicides before and during the pandemic may have affected these results. Additionally, given that the observation period in this study spanned the pre‐pandemic to the early pandemic periods, the study may have only indicated conditions before the effects of economic problems caused by the COVID‐19 pandemic became apparent.

This study has several limitations. The data used in the study were collected by a single physician in a limited area and do not reflect the actual situation in Japan as a whole. Since the analyses in this study were only exploratory, including post‐hoc analyses, and the number of cases was small, the reliability of the adjusted odds ratio and 95% confidence interval obtained from logistic regression analysis may not be high. In addition, the information that was collected from postmortem examinations is limited and does not consider factors—including sequentiality—other than those discussed in this study. Furthermore, many study variables were extracted a posteriori from free text data; therefore, their frequencies may have been underestimated.

Despite these limitations, to the best of our knowledge, this is the first study to examine the factors associated with suicide during the COVID‐19 pandemic in Japan by comparing primary data on suicide cases before and during the COVID‐19 pandemic.

We further consider that this study presented the new methodological possibility of analyzing background factors of suicide using postmortem examination data. Each research design that examines the background factors of suicide has its own strengths and weaknesses. For example, secondary statistical data published by public agencies are suitable for characterizing the total number of cases; however, analyzing detailed background information on each case is difficult with these data. In contrast, a survey of people who have experienced self‐harm or have attempted suicide has the advantage of generating subjective data from the persons themselves. However, problems such as the fact that the profile of the surveyed person (e.g., sex and characteristics of mental illness) differs from that of those who died by suicide have been highlighted.^25^, ^26^ To improve suicide prevention measures, handling data on cases of death by suicide in research and obtaining detailed background information on each death are important. In this respect, the postmortem examination data used in this study are considered more useful than secondary statistical data or data collected from suicide attempters. Although psychological autopsy studies offer more detailed background information on persons who died by suicide than postmortem examinations, the use of postmortem examination data provides the significant advantages of eliminating the recall bias of the bereaved family and allowing the collection of a large number of data with a small time lag from the occurrence of suicide. In the light of the above, analysis using postmortem examination data may be a realistic and relatively well‐balanced methodology for analyzing the background factors of suicide in Japan.

In the future, it is necessary to develop a system for continuous nationwide monitoring that combines methods such as the psychological autopsies of suicides to ensure that the more precise and real‐time analysis of data on suicides, such as that in this study, can be conducted in times of emergency.

## AUTHOR CONTRIBUTIONS

All authors were personally and actively involved in substantive work on the report and hold themselves jointly and individually responsible for its content. Yotaro Katsumata (first author) developed the original idea for the study. All authors participated in the design and coordination of the study. Toshiaki Okano (second author) collected data. Tadashi Takeshima (third author) and Yuka Igarashi (fourth author) managed data for analysis. Yotaro Katsumata performed the statistical analysis and drafted the manuscript. All authors read and approved the final manuscript. Permission was obtained from all co‐authors for submission.

## CONFLICT OF INTEREST STATEMENT

The authors declare no conflict of interest.

## ETHICS APPROVAL STATEMENT

The study was approved by the ethics committee of Tokyo Metropolitan University (H4‐63). Data were shared with the co‐researcher in accordance with national ethical guidelines (the Ethical Guidelines for Medical and Health Research Involving Human Subjects) after an opt‐out procedure that was used with the surviving family members at the clinic where the second author practices. The study results were published in accordance with the guidelines of the Japanese Society of Legal Medicine.

## PATIENT CONSENT STATEMENT

Not applicable.

## CLINICAL TRIAL REGISTRATION

Not applicable.

## Data Availability

Data cannot be shared for privacy or ethical reasons.
